# Establishment of Infection Models in Zebrafish Larvae (*Danio rerio*) to Study the Pathogenesis of *Aeromonas hydrophila*

**DOI:** 10.3389/fmicb.2016.01219

**Published:** 2016-08-04

**Authors:** Paolo R. Saraceni, Alejandro Romero, Antonio Figueras, Beatriz Novoa

**Affiliations:** Immunology and Genomics, Institute of Marine Research (IIM) – Consejo Superior de Investigaciones Científicas (CSIC), VigoSpain

**Keywords:** zebrafish larvae, infection models, *Aeromonas*, fluorescent imaging, microinjection, bath infection, immune response

## Abstract

*Aeromonas hydrophila* is a Gram-negative opportunistic pathogen of fish and terrestrial animals. In humans, *A. hydrophila* mainly causes gastroenteritis, septicaemia, and tissue infections. The mechanisms of infection, the main virulence factors and the host immune response triggered by *A. hydrophila* have been studied in detail using murine models and adult fish. However, the great limitation of studying adult animals is that the animal must be sacrificed and its tissues/organs extracted, which prevents the study of the infectious processes in the whole living animal. Zebrafish larvae are being used for the analysis of several infectious diseases, but their use for studying the pathogenesis of *A. hydrophila* has never been explored. The great advantage of zebrafish larvae is their transparency during the first week after fertilization, which allows detailed descriptions of the infectious processes using *in vivo* imaging techniques such as differential interferential contrast (DIC) and fluorescence microscopy. Moreover, the availability of fluorescent pathogens and transgenic reporter zebrafish lines expressing fluorescent immune cells, immune marker genes or cytokines/chemokines allows the host–pathogen interactions to be characterized. The present study explores the suitability of zebrafish larvae to study the pathogenesis of *A. hydrophila* and the interaction mechanisms between the bacterium and the innate immune responses through an infection model using different routes for infection. We used an early-embryo infection model at 3 days post-fertilization (dpf) through the microinjection of *A. hydrophila* into the duct of Cuvier, caudal vein, notochord, or muscle and two bath infection models using 4 dpf healthy and injured larvae. The latter resembled the natural conditions under which *A. hydrophila* produces infectious diseases in animals. We compared the cellular processes after infection in each anatomical site by confocal fluorescence imaging and determined the implication of inflammatory immune genes by measuring gene expression by qPCR.

## Introduction

Although zebrafish (*Danio rerio*) have been used in science since the 1970s (Grunwald and Eisen, 2002), only in the last decade have they become a relevant model to study human diseases such as cancers, cardiovascular and muscular malformations, and depressive disorders (Lieschke and Currie, 2007; Goldsmith and Jobin, 2012; Santoriello and Zon, 2012; Phillips and Westerfield, 2014; Lin et al., 2016). Moreover, zebrafish have been used for studying animal and human infectious disease (Novoa and Figueras, 2012; Nowik et al., 2015) because its immune system is similar to that of mammals (Dios et al., 2010; Lugo-Villarino et al., 2010; Renshaw and Trede, 2012; Van der Vaart et al., 2012). The immune system of adult zebrafish includes almost all lymphoid organs and immune cell types (Trede et al., 2004; Levraud et al., 2008; Renshaw and Trede, 2012). At early developmental stages, up to approximately 2 weeks post-fertilization, only innate immunity is active, mainly comprising macrophages and neutrophils (Herbomel et al., 1999; Lieschke et al., 2001; Lam et al., 2004; Le Guyader et al., 2008). Because of its low cost and easy large-scale breeding, zebrafish is a convenient animal model compared with rodents for identifying the roles of new genes involved in disease processes and for high-throughput applications such as drug screening (Pardo-Martin et al., 2013; Huiting et al., 2015; MacRae and Peterson, 2015; Ordas et al., 2015).

Using zebrafish, infectious processes can be described in detail using *in vivo* imaging techniques because of their small size and transparency during the first week after fertilization. These techniques primarily include differential interferential contrast (DIC) and fluorescence microscopy (O’Toole et al., 2004; Hall et al., 2009; Meijer and Spaink, 2011; Bernut et al., 2015). The availability of fluorescent pathogens and transgenic reporter zebrafish lines expressing fluorescent immune cells, immune marker genes or cytokines/chemokines allows the host–pathogen interactions to be described in detail (Benard et al., 2012; Tobin et al., 2012; Meijer et al., 2014; Torraca et al., 2014). Zebrafish larvae have been used to analyze the innate immune response after bacterial infections such as *Mycobacterium marinum* (Lesley and Ramakrishnan, 2008; Volkman et al., 2010; Adams et al., 2011; Meijer, 2016), *Streptococcus* sp. (Neely et al., 2002), *Salmonella typhimurium* (van der Sar et al., 2003), *Staphylococcus aureus* (Benard et al., 2012; Li and Hu, 2012) and *Burkholderia cenocepacia* (Vergunst et al., 2010; Mesureur and Vergunst, 2014).

Important new insights into human infectious diseases mechanisms have been uncovered by these infection models. *Mycobacterium marinum* is closely related to the global human pathogen *Mycobacterium tuberculosis* (Meijer, 2016). In zebrafish, *M. marinum* induces the formation of granulomas, which are the typical histopathological hallmark of human tuberculosis, so the *M. marinum* infection model is used to study human tuberculosis. This model highlights the fact that the stabilization of the transcription factors hypoxia-inducible factor α (HIF-α) favors the host response against *M. marinum*; the CXCR3/CXCL11 signaling axis is involved in macrophage recruitment and dissemination of mycobacterial infection, and Dram-1-mediated autophagy is an important host defense to counteract mycobacterium infection (Elks et al., 2013; Torraca et al., 2015; Meijer, 2016). These findings represent a potential target for therapeutic intervention against tuberculosis (Meijer, 2016; Myllymäki et al., 2016). The infection of zebrafish larvae with *Burkholderia cenocepacia*, which is an opportunistic pathogen of cystic fibrosis or immunocompromised individuals, revealed that this pathogen survives within macrophages, where it can replicate, and thereafter disseminates to produce fatal bacteraemia and that a functional CepIR quorum-sensing system is required for intracellular replication and dissemination (Vergunst et al., 2010). *Salmonella typhimurium* infections highlight the importance of the mitochondria-associated enzyme immunoresponsive gene 1 (IRG1) in the intracellular degradation of phagocytosed bacteria. This enzyme, which is expressed by macrophages, directs the mitochondrial catabolism of fatty acids for the production of mitochondrial ROS, contributing to the clearance of intracellular bacteria (Hall et al., 2013). This result underlines the importance of the connection between metabolism and immunity for the study of infectious diseases and highlighted IRG1 as a new therapeutic target for intracellular bacterial infections (Hall et al., 2013). Other bacterial infection models in zebrafish, such as *Pseudomonas aeruginosa*, *Edwardsiella tarda*, and *Listeria monocytogenes*, have been used to determine the pathogenicity-related factors and resistance strategies of the host (Pressley et al., 2005; Brannon et al., 2009; Levraud et al., 2009). Nevertheless, the use of zebrafish larvae for the study of the pathogenesis of *A. hydrophila* has never been explored.

The Gram-negative bacterium *Aeromonas hydrophila* is a motile, rod-shape, facultative anaerobic bacterium that is an opportunistic pathogen of fish and terrestrial animals (Janda and Abbott, 2010; Igbinosa et al., 2012). In fish, it causes motile aeromonas septicaemia (MAS), a disease that leads to a high mortality (Harikrishnan and Balasundarama, 2005). In humans, *A. hydrophila* can cause gastroenteritis, septicaemia, tissue infections and other, less frequent complications, such as peritonitis, endocarditis, pancreatic infections, and urinary tract infections (Janda and Abbott, 2010). Clinical conditions such as cancer, hepatic diseases, diabetes and trauma increase the risk to develop a fatal *A. hydrophila* infection (Parker and Shaw, 2011). The pathogenicity of *A. hydrophila* is multifactorial, depending on several virulence factors: enterotoxins (e.g., Act, Ast and Alt), haemolysins (α and β), Shiga toxins, extracellular enzymes such as proteases and nucleases, type 3 and type 6 secretion systems (T3SSs, T6SSs) and motility factors such as lateral and polar flagella (Alperi and Figueras, 2010; Tomás, 2012). Although *A. hydrophila* is naturally present in the gut microbiota of zebrafish (Cantas et al., 2012b), it is able to generate an acute infection in adults (Rodríguez et al., 2008), but no information is available about the pathogenic process and host immune response in embryos.

Numerous studies have been conducted to identify the mechanisms of infection, the main virulence factors and the host immune response triggered by *A. hydrophila* in murine and adult fish models (Yu et al., 2004, 2005; Sha et al., 2005; Canals et al., 2006, 2007; Vilches et al., 2007; Rodríguez et al., 2008, 2009; McCoy et al., 2010; Cantas et al., 2012a; Lü et al., 2015). Therapeutic strategies such as immunomodulatory molecules and vaccines have been developed in some fish species (carp, zebrafish, and rainbow trout) to protect the animals against *A. hydrophila* infection (Rodríguez et al., 2009; Poobalane et al., 2010; Bastardo et al., 2012; Brogden et al., 2012; Cao et al., 2012; Liu et al., 2015). Mice and adult zebrafish have been experimentally infected by intramuscular or intraperitoneal injections (Sha et al., 2005; Yu et al., 2005; Rodríguez et al., 2008, 2009; Chen et al., 2014), and orally by gavage or exposing the zebrafish to the bacteria in the water (Wong et al., 1996; Abuelsaad et al., 2014; Lü et al., 2015). The infectious process has been evaluated by measuring the mortality rates, bacterial burden, respiratory burst, immune gene expression, quantification of leukocytes by flow cytometry, and tissue damage by histological assays (Fadl et al., 2007; Sha et al., 2007; Suarez et al., 2010; Li and Hu, 2012). The great limitation of those methods is that they require the sacrifice of the animals and the extraction of tissues/organs to perform all of the analyses, thereby preventing the study of the infectious processes in the whole living animal.

To overcome this limitation, we chose zebrafish larvae to study *Aeromonas hydrophila* infections. The exceptional transparency of zebrafish larvae allowed us to follow the infection process without killing the animal through non-invasive *in vivo* imaging of individual cells and host–pathogen interactions at high resolution. Pathogens, immune cells and immune factors can be visualized in three-dimensional tissue architecture evidencing tissue/organs specific immune responses. In this way, it is possible to image the phagocytic activity of host immune cells and the interactions between host cells and microbe (Meijer et al., 2014). These useful features of the zebrafish model coupled with the possibility to simulate the natural mode of *A. hydrophila* infections, i.e., by bath infections, would allow the evaluation of the exact contribution of each bacterial virulence factor to *A. hydrophila* pathogenesis. By using *A. hydrophila* mutants for different virulence factors, it could be possible uncover the specific role of each in the *A. hydrophila* diseases. In addition, the high number of animals available for experiments permits the performance of high-throughput screenings of virulence (Westerfield, 2007; Spaink et al., 2013).

Thus, the starting hypothesis of the present study is that the zebrafish larvae are a suitable animal model for the study of the pathogenesis of *A. hydrophila* and the interaction mechanisms between the bacterium and the innate immune system. We exposed zebrafish to infection by three different routes. First was an early-embryo infection model at 3 days post-fertilization (dpf) through the microinjection of *A. hydrophila* into the duct of Cuvier, caudal vein, notochord, or muscle. The other two were bath infection models using 4 dpf healthy and injured larvae. The bath infection simulated the natural conditions by which *A. hydrophila* produces infectious diseases in animals. The duct of Cuvier is a wide blood circulation valley on the yolk sac where the anterior and posterior cardinal vessels join (Kimmel et al., 1995). Because the duct of Cuvier connects the heart to the trunk vasculature, it has been used to introduce pathogens such as *Staphylococcus aureus*, *Pseudomonas aeruginosa*, *Mycobacterium marinum*, and *Salmonella typhimurium* into the blood circulation by microinjection (Clatworthy et al., 2009; Cui et al., 2011; Benard et al., 2012; Li and Hu, 2012). The caudal vein is another frequent route to inject pathogens into the bloodstream to produce systemic infections (Cui et al., 2011). It has been used for pathogens such as *Mycobacterium marinum* (Meijer, 2016), *Salmonella typhimurium* (van der Sar et al., 2003), *Burkholderia cenocepacia* (Mesureur and Vergunst, 2014), *Staphylococcus aureus* (Li and Hu, 2012), *Pseudomonas aeruginosa* (Brannon et al., 2009), *Streptococcus pneumoniae* (Rounioja et al., 2012), and *Listeria monocytogenes* (Levraud et al., 2009). Notochord and muscle are alternative injection routes that are used to produce local infections (Cui et al., 2011; Benard et al., 2012). Notochordal infections have been performed to administer pathogens such as *Escherichia coli* (Nguyen-Chi et al., 2014) and *Mycobacterium marinum* (Alibaud et al., 2011), and muscle injections have been performed to study microbes such as *Salmonella typhimurium* (Zakrzewska et al., 2010), *Escherichia coli* (Nguyen-Chi et al., 2014), and *Francisella* spp. (Brudal et al., 2014).

It is critical to establish the correct infection parameters because the site, timing, and dose of the microinjection of bacteria into the embryo are important factors that determine the bacterial infection of the host (Benard et al., 2012). We observed the cellular processes after infection in each anatomical site by confocal fluorescence imaging and determined the implication of inflammatory immune genes by measuring gene expression by qPCR.

## Materials and Methods

### Ethics Statement

Fish care and experiments were performed according to EU guidelines^1^. All of the protocols were revised and approved by the CSIC Spanish National Committee on Bioethics (151/2014).

### Fish

The zebrafish (*Danio rerio*) lines used in the experiments were the wild type AB variety and the homozygous transgenic lines *Tg(mpx:GFP+/+)* and *Tg(il-1b:GFP-F+/+)* (Renshaw et al., 2006; Nguyen-Chi et al., 2014). Mixed male and female populations of zebrafish were kept in 9-liter tanks at 28°C. Embryos were obtained by natural spawning and reared in E3 egg water for 3 days until hatching (Westerfield, 2007). Embryos at 3 and 4 dpf were used in the experiments.

### Bacteria

The *Aeromonas hydrophila* AH-1 strain (Yu et al., 2004) and the fluorescent *A. hydrophila* Ds-Red (kindly provided by Dr. Van der Sar, The Netherlands) were used. For experimental infections, the bacteria were grown on tryptic soy agar (TSA) plates overnight at 28°C and were resuspended in phosphate-buffered saline (PBS) to obtain a stock solution containing 10^10^ colony-forming units (CFUs)/mL (OD_620nm_ = 1.3). Dilutions to the desired concentration were prepared for the different experimental infections.

### Infection Models

Two different routes of bacterial inoculation were assayed. A microinjection model was used to produce local or systemic infections depending on the anatomical site of injection. Two bath infection models were used, in which the animals were exposed to the bacteria in the water. The two bath infection models were immersion only and immersion after injury, in which the animals were injured in the tail fin tip to generate an alternative portal of entry to the bacteria.

#### Microinjection Model

AB zebrafish larvae at 3 dpf were anesthetized in E3 eggs water containing 160 μg/ml MS-222 (Sigma–Aldrich), placed on an agarose plate and individually microinjected using pulled glass microcapillary pipettes (WPI, USA). Larvae were microinjected with 1 nL of an *A. hydrophila* solution (3200 CFU/nL) into the duct of Cuvier, caudal vein, notochord, or muscle, following the recommendations described by Benard et al. (2012). The first control group was injected with the same volume of PBS in the duct of Cuvier. The second control group was injected with the same volume of dead *A. hydrophila* to show that the death of larvae was not simply caused by a general process in response to the infection. Microinjection was performed using the micromanipulator Narishige IM-30 and the stereo microscope SMZ800 (Nikon). In all experiments the control group was injected with the same volume of PBS. The exact bacterial concentration injected into the animals was determined by plating dilutions of the injected solution onto LB agar and counting the obtained CFUs after overnight incubation at 28°C. Infected and control larvae were maintained at 28°C in six wells plate (Falcon) containing 6 ml of E3 egg water. Animals were regularly observed, and cumulative mortalities were registered until 5 days post-infection (dpi). Three independent experimental infections were conducted. A total of 40 animals (four biological replicates of 10 larvae each) were infected through each anatomical site.

The duct of Cuvier was selected for additional experimental infections. Larvae were microinjected with 1 nL of an *A. hydrophila* dilution (3175 CFU/nL, 1150 CFU/nL, or 375 CFU/nL). Three independent experimental infections were conducted. A total of 40 animals (four biological replicates of 10 larvae each) were infected in each trial.

#### Bath Infection Model

AB zebrafish larvae (healthy) at 4 dpf were used for bath immersion infections. Two models were used: immersion only and immersion after injury, in which the animals were injured in the tail fin tip before the infection. Experimental infections of healthy and injured larvae were performed in parallel using the same bacterial suspension at the same concentration. To produce a wound in larvae, animals were anesthetized and placed on a Petri dish filled with 1% low-melting-point agarose (Sigma–Aldrich). A small transection of the tail fin was done using a sapphire blade (WPI) under the stereomicroscope SMZ800 (Nikon).

Groups of 10 healthy and injured larvae were distributed into 6-well plates (Falcon) containing 6 ml of sterile E3 eggs’ water. For the infection, *A. hydrophila* stock solution was added to each well to reach a final concentration of 10^8^ CFU/mL, and the plates were incubated at 28°C. Injured larvae were immediately immersed in the bacterial solution after the transection of the tail. The inoculated bacterium was kept in the water throughout the experiment. The control groups (injured and healthy larvae) were treated with PBS. Cumulative mortalities were registered until 6 dpi. The experimental infections were performed 5 times using four biological replicates of 10 larvae each.

### Bacterial Load in Fish during Infection

The bacterial load in fish was measured after microinjection (0 and 24 hpi) or bath infection (1, 6, and 24 hpi). Four groups of 10 microinjected larvae and the same number of bath-infected larvae were anesthetized with a lethal dose of MS-222 (Sigma–Aldrich), transferred into a tube containing 200 μl of 1% Triton-X100 (BIO-RAD) and mechanically homogenized. Serial dilutions of the homogenates were prepared in PBS and plated in selective TSA plates. CFUs were counted after overnight incubation at 28°C in two independent experiments.

### *In vivo* Characterization of the Cellular Immune Response by Fluorescence Microscopy

The homozygous transgenic zebrafish line *Tg(mpx:GFP+/+)*, expressing fluorescent neutrophils, was used to visualize the response of neutrophils to the infection (Renshaw et al., 2006). The homozygous transgenic zebrafish line *Tg(Il-1b:GFP-F+/+)*, expressing GFP under the control of the Il-1β promoter was used to visualize the expression of Il-1β gene (Nguyen-Chi et al., 2014). In all experiments, *N*-phenylthiourea 0.2 mM (PTU; Sigma) was used to prevent pigment formation.

Larvae at 3 dpf were injected with 1 nL of an *A. hydrophila* suspension in the different anatomical sites as described above. The bacterial suspension injected in the *Tg(mpx:GFP+/+)* and *Tg(Il-1b:GFP-F+/+)* contained 5600 CFU/nL and 1400 CFU/nL, respectively. The first control group was injected with the same volume of PBS in the duct of Cuvier. The second control group was injected with the same volume of dead *A. hydrophila.* At 6 hpi, animals were fixed in 4% paraformaldehyde, washed in PBS-Tween 0.1% (Sigma–Aldrich) and imaged in a TSC SPE confocal microscope (Leica) using a 10X HCX APO (L 0.30 W U-V-I) water objective (Leica). Additional infections of *Tg(mpx:GFP+/+)* larvae by injection in the duct of Cuvier (1 nL of a bacterial suspension containing 200 CFU/nL) were conducted and imaged using a TSC SPE confocal microscope (Leica). The total neutrophils were counted at different times post-infection (2, 4, 6, and 24 h) using the ImageJ software (Ellett and Lieschke, 2012). Neutrophils were counted in two independent experiments using 10 larvae at each time point.

The involvement of neutrophils was also evaluated in 4 dpf healthy and injured *Tg(mpx:GFP+/+)* larvae at 1 h after bath infection. Animals were infected with *A. hydrophila* DsRed (at a final concentration of 10^8^ CFU/mL), and the neutrophils in the injured area were counted as above using the TSC SPE confocal microscope (Leica) and ImageJ. Neutrophils were counted in three independent experiments using 10–15 larvae. Z-stacks images were used for 3D reconstructions using the Image Surfer software (Feng et al., 2007).

### qPCR

The expression of the proinflammatory genes TNFα and IL-1β was evaluated in AB larvae after bath exposure or microinjection in the duct of Cuvier. Healthy and injured larvae (*n* = 30 fish/group) were bath-infected with 10^8^ CFU/mL *Aeromonas* and sampled at 1, 3, and 5 hpi. For microinjection in the duct of Cuvier, 30 larvae were injected with 1 nL of *A. hydrophila* (120 CFU/nL) and sampled at 6 hpi. In both experimental infections, three biological replicates, 10 larvae per replicate, were sampled in each sampling point and suspended in 200 μl lysis buffer (Promega). Total RNA was isolated using the Maxwell^®^ 16 LEV simply RNA Tissue kit (Promega) according to the manufacturer’s instructions. First-strand cDNAs were synthesized using SuperScript II (Life Technologies) from 500 μg of total RNA per sample. Specific qPCR primers were designed using the Primer3 software (Rozen and Skaletsky, 2000) (**Table 1**). The efficiency of the primer pairs was analyzed with seven serial fivefold dilutions of cDNA and calculated from the slope of the regression line of the cycle thresholds (Cts) versus the relative concentration of cDNA (Pfaﬄ, 2001).

**Table 1:** Sequence of primers used for qPCR experiments.

Gene	Primer	Sequence (5′–3′)	Amplicon (bp)	Efficiency
EF1α	Forward	GCATACATCAAGAAGATCGGC	121	-3.45
	Reverse	TCTTCCATCCCTTGAACCAG		
TNFα	Forward	ACCAGGCCTTTTCTTCAGGT	148	-3.38
	Reverse	GCATGGCTCATAAGCACTTGTT		
IL-1β	Forward	TTCCCCAAGTGCTGCTTATT	149	-3.33
	Reverse	AAGTTAAAACCGCTGTGGTCA		

qPCR was carried out in a 7300 Real Time PCR System (Applied Biosystems) using standard cycling conditions. One microliter of cDNA was mixed with 0.5 μl of each primer (final concentration 10 mM), 12.5 μl of SYBR green PCR master mix (Applied Biosystems) and 10.5 μl of water. The elongation factor 1-alpha gene (EF1α) was used as the normalization gene because it was constitutively expressed and not affected by the bacterial infection. The relative expression levels of TNFα and IL-1β were normalized using the expression levels obtained for the EF1α gene. Fold units were calculated by dividing the normalized expression values obtained in infected samples by the normalized expression values obtained in the control at each sampling point (Pfaﬄ, 2001). The results of three independent experimental infections were expressed as the mean ± standard error.

### Statistical Analysis

All data were analyzed using Student’s *t*-test (two-tailed, unpaired). The results are presented as the means ± SEM. *P* < 0.05 (*); *P* < 0.01 (**); and *P* < 0.005 (***) were considered significant. Multiple-comparison ANOVA and the Tukey HSD test were conducted to evaluate the variations in the number of neutrophils and the evolution of the gene expression.

## Results

### Development of the Microinjection Model

**FIGURE 1:**
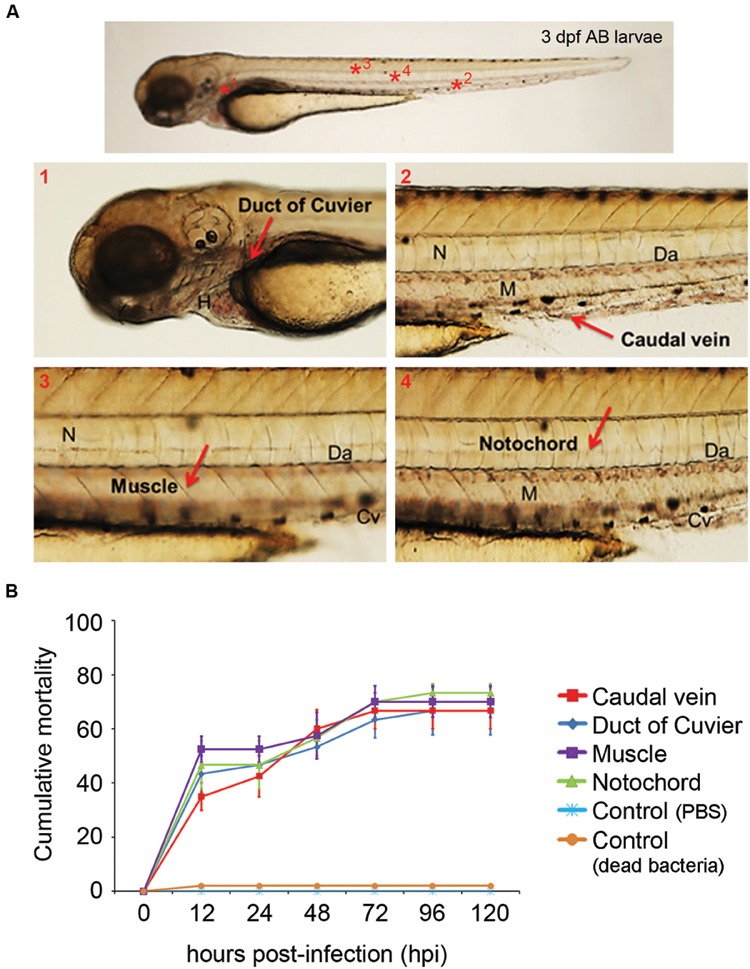
**(A)** Anatomical distribution of the selected sites for microinjection. The duct of Cuvier (A1) and the caudal vein (A2) were selected to establish systemic infections. Injection in the muscle (A3) or notochord (A4) induced a local infection. Cv, caudal vein; Da, dorsal aorta; M, muscle; N, notochord. **(B)** Evolution of cumulative mortality in larvae infected with 1 nL of an *A. hydrophila* solution (3200 CFU/nL). The graph shows a representative result of the three independent experiments conducted. Forty larvae were injected in each anatomical site. No significant differences were observed between the mortality registered using the different injection sites throughout the experiment.

The microinjection of *A. hydrophila* in 3 dpf larvae generated two different types of infections according to the selected anatomical site. Injection in the duct of Cuvier or in the caudal vein generated a systemic infection (**Figure 1**, panels A1 and A2), and injection in the muscle or notochord induced a local infection (**Figure 1**, panels A3 and A4).

The experimental infection of larvae with *A. hydrophila* (3200 CFU/nL) induced elevated mortality regardless of the site selected for the injection. In both local and systemic infections, mortality started as soon as 12 hpi and reached a 65–73% cumulative mortality at 96 hpi. In all cases, no significant differences in cumulative mortality were registered between systemic and local infections. No mortalities were registered in fish injected with the same volume of PBS or dead *A. hydrophila* (**Figure 1B**).

**FIGURE 2:**
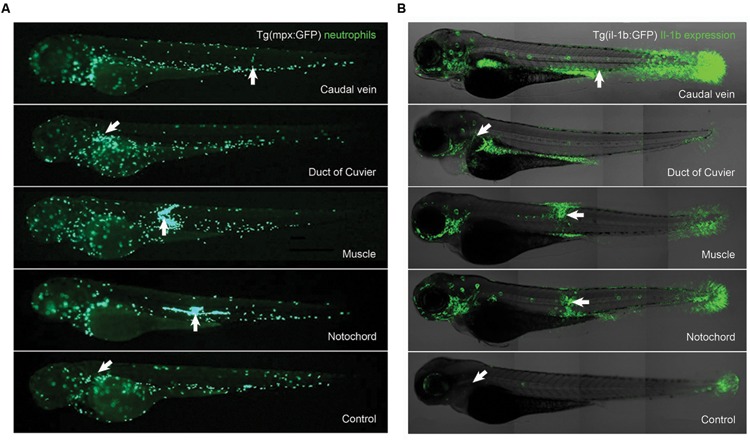
**(A)** *In vivo* imaging of neutrophil migration after infection of *Tg(mpx:GFP+/+)* larvae with *A. hydrophila*. Larvae were injected with 1 nL of *A. hydrophila* (5600 CFU/nL) and imaged at 6 hpi. Images show differential migration of neutrophils depending on the anatomical site of injection. **(B)** Anatomical distribution of IL-1β gene expression in larvae microinjected at different anatomical sites. *Tg(il-1b:GFP+/+)* larvae were injected with 1 nL of *A. hydrophila* (1400 CFU/nL) and imaged at 6 hpi. Overlay of IL1-β expression (green), with the transmission channel used as an anatomical guide. The white arrows indicate the positions of the injection sites.

The experimental infection of the *Tg(mpx:GFP+/+)* larvae allowed us to characterize the *in vivo* response of neutrophils to the bacteria by fluorescence microscopy (**Figure 2A**). A strong migration of neutrophils to the injection area was clearly observed after 6 hpi in the muscle and the notochord (**Figure 2A**). At this time, the injection in the duct of Cuvier induced less neutrophil migration compared to that observed in the muscle or notochord. The injection in the caudal vein did not induce local migration to the site of injection (**Figure 2A**). The injection in the duct of Cuvier or caudal vein induced a general increase in the number of neutrophils compared to the PBS-injected control group (**Figure 2A**).

The *Tg(il-1b:GFP-F+/+)* larvae were also used to visualize the anatomical distribution of Il-1β expression after the infection in the different sites. Different tissue localization of Il-1β expression was observed depending on the injection route (**Figure 2B**). Larvae microinjected in the muscle or in the notochord showed strong IL-1β expression around the injection site and also in the gills and the tail fin. Larvae injected in the duct of Cuvier or caudal vein presented the strongest IL-1β expression along the intestine. Larvae injected in the caudal vein showed elevated IL-1β expression in the tail fin. The expression observed in the gills was lower than that observed in larvae injected in the muscle or notochord (**Figure 2B**). In all cases the distribution of the IL-1β expression after infection was highly representative because 80% of the larvae injected in the same site presented similar GFP distribution.

Larvae injected with the dead bacteria in the duct of Cuvier showed a neutrophil distribution and IL-1B expression similar to that observed in PBS injected larvae (data not shown).

#### Infection Model by Injection in the Duct of Cuvier

The injection in the duct of Cuvier was selected for additional experiments because this route was the easiest to generate a quick systemic infection and because it induced a local inflammatory reaction. With these experiments, we developed a more detailed description of mortality kinetics and the induction of immune response.

**FIGURE 3:**
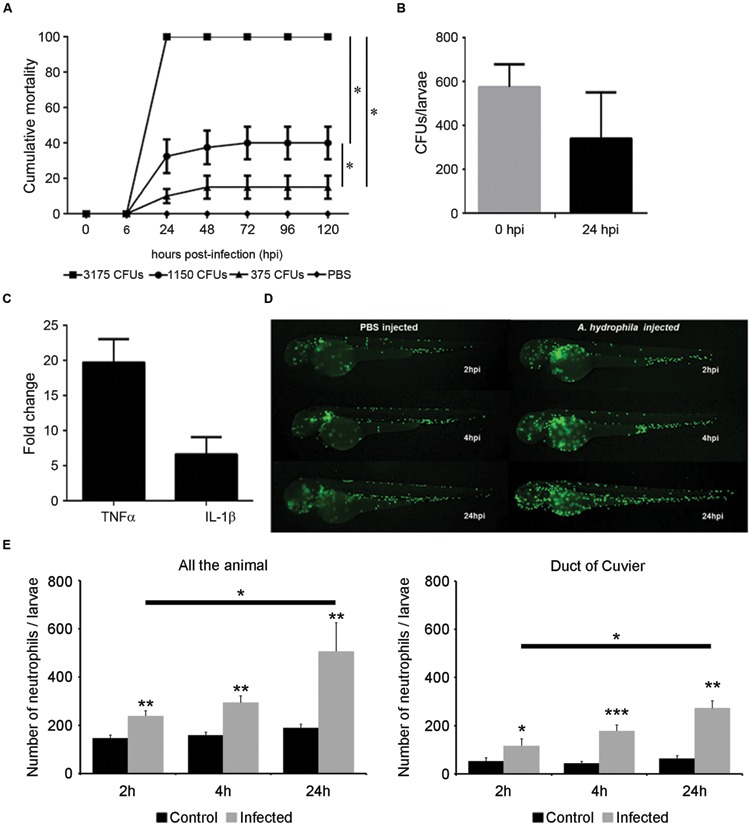
**Infection model by microinjection in the duct of Cuvier. (A)** Cumulative mortality in larvae injected with various doses of bacteria (3175 CFU/nL, 1150 CFU/nL, and 375 CFU/nL). Graph shows a representative experiment of three independent infections (*n* = 40 larvae per group). The dilution of the bacteria induced a significant decrease of mortality (*P < 0.05). **(B)** Bacterial burden of infected larvae at 0 and 24 hpi. A representative result (mean ± SEM) of two independent experiments is presented (*n* = 40 larvae per group). **(C)** Increased TNFα and IL1-β expression in infected larvae at 6 hpi (*n* = 30 larvae per group). **(D)** *In vivo* response of neutrophils after infection. *Tg(mpx:GFP+/+)* larvae were injected with *A. hydrophila* (200 CFU/nL) and imaged at 2, 4, and 24 hpi. **(E)** Total neutrophil counts in infected and control larvae in all the body (systemic infection) and in duct of Cuvier (local infection). Results represent the mean ± SEM of two independent experiments (*n* = 10 larvae per group; ANOVA and Tukey HSD test; *P < 0.05, **P < 0.01; ***P < 0.005).

The injection of 3175 CFU/nL induced mortality rates similar to those obtained in previous experiments, reaching 100% cumulative mortality at the end of the experiment (**Figure 3A**). The dilution of the bacteria induced a significant decrease in mortality (less than 20% at the end of the experiment; **Figure 3A**). The evolution of the bacterial burden was analyzed to evaluate whether the microinjection in the duct of Cuvier induced a stable bacterial infection. The bacterial burden of viable bacteria did not change significantly from 0 to 24 hpi (575 and 340 CFU at 0 and 24 hpi, respectively; **Figure 3B**).

The suitability of this infection model to the study of the immune response was analyzed by determining the expression of proinflammatory genes and by characterizing the response of neutrophils. Six hours after the injection of *A. hydrophila*, the expression of the TNFα gene in infected larvae was up to 20 times higher than the control larvae. The expression of the IL-1β gene was also increased but was only five times that of the control group (**Figure 3C**). The injection of *Tg(mpx:GFP+/+)* larvae revealed a fast recruitment of neutrophils around the injection site short time after infection (2 and 4 hpi; **Figure 3D**). A significant increase of total neutrophils was registered in all the body (systemic response) and in the injection site (local response) in all infected animals compared to the control groups, regardless of the sampling point. Moreover, a significant increase in the number of neutrophils was observed in infected animals from 2 to 24 hpi also in all the body and in the injection site (**Figure 3E**).

### Development of the Bath Infection Model

**FIGURE 4:**
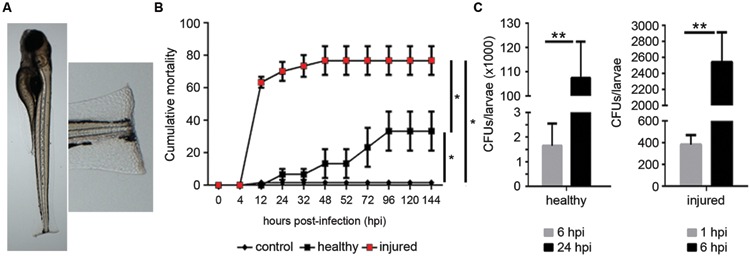
**Development of bath infection model. (A)** Larvae were injured in the tail fin tip using a sapphire blade. **(B)** Cumulative mortality of healthy and injured larvae infected with *A. hydrophila* (10^8^ CFU/mL). Fish in the control group were injured in the tail fin and treated with PBS. Graph is a representative result of five independent experiments using 40 larvae in each experiment. The injury induced a significant increase of mortality (*P < 0.05). **(C)** Bacterial burden in infected healthy and injured larvae at 1, 6, and 24 hpi. Data represent the mean ± SEM of two independent experiments (*n* = 40 larvae/each). Significant differences at **P < 0.01.

Larvae at 4 dpf were used in this model because at this developmental stage the mouth is open and the bacteria suspended in the water can enter there. The injury to the tail fin provided an alternative portal of entry (**Figure 4A**). The experimental infection of larvae with *A. hydrophila* induced mortalities in both healthy and injured larvae (**Figure 4B**). In healthy larvae, mortalities began at 12 hpi and increased during the experiment, reaching a maximum cumulative mortality of 33% (±12) at 96 hpi and staying constant until the end of the experiment (**Figure 4B**). However, the same bacterial dose inoculated in injured larvae induced a significant increase of mortality (**Figure 4B**). In injured larvae, mortalities began soon after the infection. At 12 hpi cumulative mortalities reached 63% (±3) and increased up to 77% (±9) by the end of the experiment (**Figure 4B**). A 1% cumulative mortality was registered in injured larvae treated with PBS (control group) at the end of the experiment.

To analyze the progression of the infection, the bacterial burden was calculated in the whole larvae at different times post-infection (**Figure 4C**). In healthy larvae, the number of bacteria inside the animal increased significantly from 6 to 24 hpi. The bacterial load per larva changed from 2.000 CFU at 6 hpi to 110.000 CFU at 24 h (55-fold; **Figure 4C**). In injured larvae, the bacterial burden was calculated at earlier time points than those used in healthy larvae (1 and 6 hpi) because mortalities occurred faster in this model. In these animals, the bacterial burden increased significantly from 400 CFU at 1 hpi to 2600 CFU at 6 hpi (6,5-fold; **Figure 4C**).

**FIGURE 5:**
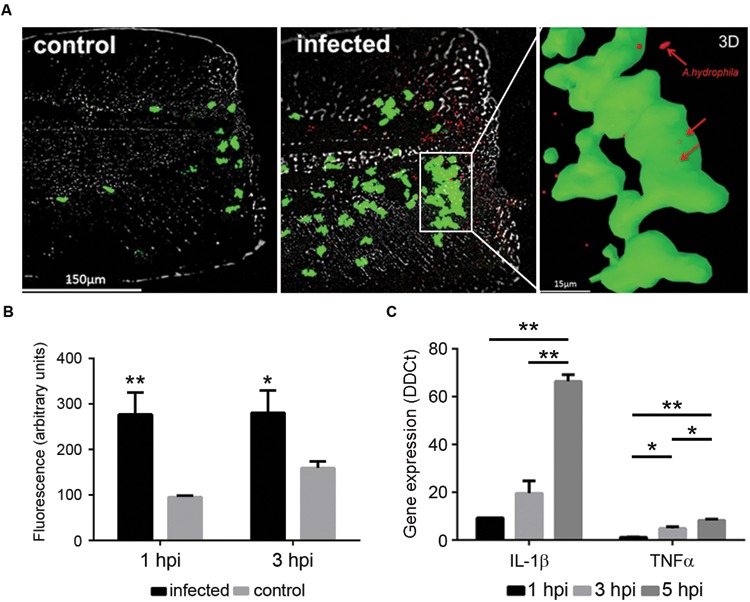
**(A)** *In vivo* imaging of neutrophils from *Tg(mpx:GFP+/+)* larvae after injury and bath infection with *A. hydrophila* (10^8^ CFU/mL). Recruitment of neutrophils to the site of the injury 1 h after the bacterial infection. Control animals were injured but not infected. 3D reconstruction of a selected site in the infected sample showing the positions of the bacteria (red) inside the neutrophils (green). **(B)** Relative quantification of neutrophils in the site of injury. Results are representative of three independent experiments (*n* = 10–15 larvae each; ANOVA; *P < 0.05, **P < 0.01). **(C)** Expression of IL-1β and TNFα in injured larvae infected by bath immersion (10^8^ CFU/mL) at 1, 3, and 5 hpi. Results of three independent experimental infections (*n* = 30) expressed as the mean ± standard error (ANOVA and Tukey HSD test; *P < 0.05, **P < 0.01).

The *Tg(mpx:GFP+/+)* larvae were infected with the fluorescent *A. hydrophila* DsRed to localize the bacteria in infected larvae and to analyze the interaction of the bacteria with the neutrophils in the site of injury. Infected animals showed a higher number of neutrophils in this area compared to the animals that were injured but not infected (**Figure 5A**). Moreover, fluorescent bacteria were present in the skin surrounding the tail fin wound (**Figure 5A**). 3D reconstructions of multiple z-stack images of the wounded sites of infected larvae showed bacteria inside of the neutrophils (**Figure 5A**). When we quantified the increase in neutrophils in the wounds of injured larvae, infected animals showed a significantly higher recruitment of neutrophils in these areas compared to the injured but uninfected animals. The number of neutrophils in the wounds of infected larvae was fairly constant until 3 hpi. Uninfected larvae presented lower neutrophil recruitment, although it increased from 1 to 3 hpi (**Figure 5B**).

The immune response triggered by the infection in injured larvae was also assayed by measuring the expression of the proinflammatory genes IL-1β and TNFα (**Figure 5C**). The infection induced a very high expression of IL-1β as soon as 1 hpi, and it significantly increased over time, reaching up to 66 times the expression detected in control animals at 5 hpi. The TNFα gene showed a similar expression pattern, although the fold change was lower, reaching a value of 8.3 at 5 hpi (**Figure 5C**).

## Discussion

In this work an infection model using different routes for infection was established in zebrafish larvae to study the pathogenesis of *A. hydrophila* and the immune response triggered in the larvae. Two infection models were proposed: an early-embryo infection model at 3 dpf through microinjection into the duct of Cuvier, caudal vein, notochord, or muscle, and two bath infection models using 4 dpf healthy and injured larvae, to simulate the natural conditions by which *A. hydrophila* produces infectious diseases.

Microinjection allows the administration of a tightly controlled bacterial load into specific anatomical sites of the larvae (Benard et al., 2012; Li and Hu, 2012). One of the most important parameters to control in this model is the bacterial dose. In our work, the injection of 3200 CFU/nL produced high mortality rates, making difficult the detection of differences between the injection sites. This observation has also been described by Li and Hu (2012), who established the infection model of *Staphylococcus aureus* and reported differences in mortalities between the injection sites only at low bacterial dose.

Different sites of infection induce different effects. Local bacterial infections were produced after an injection in the muscle or notochord, whereas an injection in the duct of Cuvier or in the caudal vein induced a systemic infection. The distinction between local and systemic infection was also evidenced by the migration of neutrophils observed in infected *Tg(mpx:GFP+/+)*. Neutrophils migrate to the infection sites to phagocytose pathogens, to release antibacterial molecules from their granules and to regulate the inflammatory response by producing cytokines and other inflammation mediators (Mantovani et al., 2011; Kolaczkowska and Kubes, 2013). Regarding leukocyte migration, the injection in the muscle or notochord of *A. hydrophila* induced strong recruitment of neutrophils in the larvae, similar to other studies in mice injected intramuscularly with *A. hydrophila* (Ko et al., 2005) and in zebrafish infected with other bacterial pathogens (Li and Hu, 2012; Deng et al., 2013; Nguyen-Chi et al., 2014). Interestingly, the injection of *A. hydrophila* in the caudal vein or in the duct of Cuvier induced a general increase in the number of total neutrophils. This increase in the number of neutrophils after a systemic challenge has been described using other bacterial models, such as *Shigella flexneri* and *Staphylococcus aureus* (Prajsnar et al., 2008; Mostowy et al., 2013).

The infection of the transgenic zebrafish line *Tg(Il-1b:GFP-F+/+)* allowed the visualization of Il-1β gene expression in different organs. IL-1β is a marker for inflammation because it is produced after a bacterial infection to stimulate the extravasation of leukocytes to the infected sites and to activate the production of antimicrobial effectors (Dinarello, 2009). In our work, differential anatomical distribution of the IL-1β expression was observed between local and systemic infection. The local infection caused by injection of bacteria in the muscle or notochord induced the expression of IL-1β mainly around the site of infection. This gene expression pattern has been described in local injection of zebrafish larvae with *E. coli* (Nguyen-Chi et al., 2014). The systemic infection of *A. hydrophila* induced an unexpectedly high expression of IL-1β mainly along the intestine. Moreover, increased IL-1β expression was observed in both local and systemic infections in other tissues, such as the gills and the tail fin. The expression of IL-1β in the embryo tail fin was not related to the experimental infection because GFP is constitutively expressed in *Tg(Il-1b:GFP-F+/+)* embryos after 50 h post fertilization in keratinocytes at the tip of the caudal fin and in fin buds, retina, neuromasts, gills, and thymus (Nguyen-Chi et al., 2014). Further studies are needed to clarify the role of IL-1β in the intestine and gills in response to *A. hydrophila* infection.

In the zebrafish embryo, the duct of Cuvier is considered one of the best routes for pathogen administration. It connects the cardinal veins to the sinus venosus of the heart (Isogai et al., 2001), thus allowing the injection into circulation of a definite quantity of pathogen (Clatworthy et al., 2009; Benard et al., 2012; Varela et al., 2014). By injecting through the duct of Cuvier, we generated a controlled systemic infection that was able to produce robust and reproducible mortalities in a dose-dependent manner. The injection of bacteria in the blood produced an infection without significant changes in bacterial burden. This is not surprising: although the blood is a good medium for bacteria to proliferate (Li and Hu, 2012), the ability of larvae to control the proliferation of *A. hydrophila* was probably related to the low dose used for the injections, as demonstrated previously for other bacterial species (Mostowy et al., 2013).

The inflammatory process started soon after the injection. The TNFα gene is also a key molecule of inflammatory process contributing to the activation of leukocytes and their recruitment to the infection site (Wajant et al., 2003; Bradley, 2008). In our work, high increases of TNFα and IL-1β gene expression were registered after *A. hydrophila* challenge, in accordance with previous studies in mice and adult fish (Fadl et al., 2007; Rodríguez et al., 2008). Variations in the expression of inflammatory genes were accompanied by a quick increase of total neutrophils. In the first hours post-challenge, the injection in the duct of Cuvier induced a local migration to the injection site, but at 24 hpi, a generalized increase in total neutrophils was observed. This result suggests that this infection model is suitable for studying both the early local response and the late systemic one. Methodologically, the use of the transgenic zebrafish larvae *Tg(mpx:GFP+/+)* in combination with fluorescent microscopy presents clear advantages compared to the classical quantification of leukocytes by flow cytometry in mice and adult fish (Rodríguez et al., 2008; Suarez et al., 2010; Khajanchi et al., 2011).

*Aeromonas hydrophila* is a water-borne bacterium that can infect humans through the mouth and wounds in the skin (Janda and Abbott, 2010; Behera et al., 2011). For this reason, a bath infection model resembling the natural conditions of infection was developed. Larvae at 4 dpf were used to ensure the mouth was open, and in one group, we also inflicted a small injury in the tail fin tip to promote a wound infection. Injured larvae showed a significantly faster and higher mortality rate than healthy larvae when challenged with the same concentration of *A. hydrophila*. Similar observations have been described in experimental infections with *A. hydrophila* using adult zebrafish (Rodríguez et al., 2008), thus suggesting the opportunistic nature of this bacterium (Janda and Abbott, 2010). The generation of an alternative portal of entry to the bacteria allows the development of a faster infectious process and the use of a lower bacterial dose to induce mortality levels similar to those induced in infected healthy larvae. We must note that the result could have been different if the bath infection had been performed at different times post-injury, but this should be further explored. The higher bacterial concentration required in this model compared to the microinjection model highlights the harmfulness of this bacterium when it is inside the blood circulation or tissues of animals. The increase of the bacterial burden after bath infection suggests that *A. hydrophila* was able to produce a stable infection in this model. Moreover, the higher bacterial burden recovered from injured larvae at 6 hpi indicates that wounds favored *A. hydrophila* adherence and colonization in the host and thus the disease.

Confocal microscopy analysis of *Tg(mpx:GFP+/+)* larvae infected with the fluorescent *A. hydrophila*-DsRed revealed the presence of adhered bacteria only in the wounded sites, indicating that surface injuries promoted the bacterial attachment to the skin (Beachey, 1981; Mertz et al., 1987; Benhamed et al., 2014). Several studies in zebrafish have demonstrated the migration of neutrophils to the injured sites to contribute to the inflammation process and healing (Yoo and Huttenlocher, 2011; Deng and Huttenlocher, 2012). Interestingly, a significant increase in neutrophil recruitment to the fin wound was observed after infection, suggesting that *A. hydrophila* amplified the inflammatory response to the tail. 3D reconstruction analysis revealed that zebrafish neutrophils can also phagocytose *A. hydrophila* associated to the body surface, in line with previous observations (Colucci-Guyon et al., 2011).

Tissue injuries induce an inflammatory status with the expression of IL1-β, TNFα and other proinflammatory genes (Eming et al., 2007). We found higher expression levels of IL1-β and TNFα at different times post-infection in injured larvae compared to injured but uninfected larvae. This result suggests that *A. hydrophila* can amplify the inflammatory response induced by the tail fin injury. Thus, the increased expression of IL1-β triggered by *A. hydrophila* could have enhanced the recruitment of neutrophils to the wounds in infected larvae because IL1-β regulates the recruitment of leukocytes to the injury sites (Ogryzko et al., 2014; Yan et al., 2014). All of these results show that skin injuries promote the bacterial attachment to larvae, which in turn induces a significant increase of the neutrophil recruitment to the wound and primes a very strong inflammatory response.

## Conclusion

Two infection models were established in zebrafish larvae to study *A. hydrophila* pathogenesis. Microinjection in the duct of Cuvier allows the generation of a controlled systemic infection, and bath infection in injured larvae mimics the natural routes of infection. Both models presented here will be useful for the study different aspects of *A. hydrophila* infection.

## Author Contributions

BN, AR, and AF contributed to the conception of the work. PS and AR acquired and analyzed data. PS, AR, BN, and AF interpreted the data. PS, AR, and BN draft the work. AF, AR, and BN revised it critically for important intellectual content. PS, AR, AF, and BN approve the final version of the article to be published.

## Conflict of Interest Statement

The authors declare that the research was conducted in the absence of any commercial or financial relationships that could be construed as a potential conflict of interest.

## Footnotes

1 http://ec.europa.eu/environment/chemicals/lab_animals/home_en.htm

## References

[B1] Abuelsaad A. S. Allam G. Al-Solumani A. A. (2014). Hesperidin inhibits inflammatory response induced by *Aeromonas hydrophila* infection and alters CD4+/CD8+ T cell ratio. *Mediators Inflamm.* 2014:393217 10.1155/2014/393217 PMC4033591 24891765

[B2] Adams K. N. Takaki K. Connolly L. E. Wiedenhoft H. Winglee K. Humbert O. (2011). Drug tolerance in replicating mycobacteria mediated by a macrophage-induced eﬄux mechanism. *Cell* 145 39–53. 10.1016/j.cell.2011.03.017 21376383 PMC3117281

[B3] Alibaud L. Rombouts Y. Trivelli X. Burguière A. Cirillo S. L. Cirillo J. D. (2011). A Mycobacterium marinum TesA mutant defective for major cell wall-associated lipids is highly attenuated in *Dictyostelium discoideum* and zebrafish embryos. *Mol. Microbiol.* 80 919–934. 10.1111/j.1365-2958.2011.07618.x 21375593

[B4] Alperi A. Figueras M. J. (2010). Human isolates of *Aeromonas* possess Shiga toxin genes (stx1 and stx2) highly similar to the most virulent gene variants of *Escherichia coli*. *Clin. Microbiol. Infect.* 16 1563–1567. 10.1111/j.1469-0691.2010.03203.x 20219084

[B5] Bastardo A. Ravelo C. Castro N. Calheiros J. Romalde J. L. (2012). Effectiveness of bivalent vaccines against *Aeromonas hydrophila* and *Lactococcus garvieae* infections in rainbow trout *Oncorhynchus mykiss* (Walbaum). *Fish Shellfish Immunol.* 32 756–761. 10.1016/j.fsi.2012.01.028 22326941

[B6] Beachey E. H. (1981). Bacterial adherence: adhesin-receptor interactions mediating the attachment of bacteria to mucosal surface. *J. Infect. Dis.* 143 325–345. 10.1093/infdis/143.3.325 7014727

[B7] Behera B. Bhoriwal S. Mathur P. Sagar S. Singhal M. Misra M. C. (2011). Post-traumatic skin and soft tissue infection due to *Aeromonas hydrophila*. *Indian J. Crit. Care Med.* 15 49–51. 10.4103/0972-5229.78228 21633548 PMC3097544

[B8] Benard E. L. van der Sar A. M. Ellett F. Lieschke G. J. Spaink H. P. Meijer A. H. (2012). Infection of zebrafish embryos with intracellular bacterial pathogens. *J. Vis. Exp.* 61:3781 10.3791/3781 PMC3415172 22453760

[B9] Benhamed S. Guardiola F. A. Mars M. Esteban M. Á. (2014). Pathogen bacteria adhesion to skin mucus of fishes. *Vet. Microbiol.* 171 1–12. 10.1016/j.vetmic.2014.03.008 24709124

[B10] Bernut A. Lutfalla G. Kremer L. (2015). Looking through zebrafish to study host-pathogen interactions. *Med. Sci. (Paris)* 31 638–646. 10.1051/medsci/20153106017 26152168

[B11] Bradley J. R. (2008). TNF-mediated inflammatory disease. *J. Pathol.* 214 149–160. 10.1002/path.2287 18161752

[B12] Brannon M. Davis J. Mathias J. Hall C. Emerson J. Crosier P. (2009). *Pseudomonas aeruginosa* Type III secretion system interacts with phagocytes to modulate systemic infection of zebrafish embryos. *Cell. Microbiol.* 11 755–768. 10.1111/j.1462-5822.2009.01288.x 19207728 PMC2933946

[B13] Brogden G. von Köckritz-Blickwede M. Adamek M. Reuner F. Jung-Schroers V. Naim H. Y. (2012). β-Glucan protects neutrophil extracellular traps against degradation by *Aeromonas hydrophila* in carp (*Cyprinus carpio*). *Fish Shellfish Immunol.* 33 1060–1064. 10.1016/j.fsi.2012.08.009 22959188

[B14] Brudal E. Ulanova L. S. O Lampe E. Rishovd A. L. Griffiths G. Winther-Larsen H. C. (2014). Establishment of three *Francisella* infections in zebrafish embryos at different temperatures. *Infect. Immun.* 82 2180–2194. 10.1128/IAI.00077-14 24614659 PMC4019159

[B15] Canals R. Jiménez N. Vilches S. Regué M. Merino S. Tomás J. M. (2006). The UDP N-Acetylgalactosamine 4-Epimerase gene is essential for mesophilic *Aeromonas hydrophila* serotype O34 virulence. *Infect. Immun.* 74 537–548. 10.1128/IAI.74.1.537-548.2006 16369010 PMC1346635

[B16] Canals R. Jiménez N. Vilches S. Regué M. Merino S. Tomás J. M. (2007). Role of Gne and GalE in the virulence of *Aeromonas hydrophila* serotype O34. *J. Bacteriol.* 189 540–550. 10.1128/JB.01260-06 17098903 PMC1797372

[B17] Cantas L. Midtlyng P. J. Sørum H. (2012a). Impact of antibiotic treatments on the expression of the R plasmid tra genes and on the host innate immune activity during pRAS1 bearing *Aeromonas hydrophila* infection in zebrafish (*Danio rerio*). *BMC Microbiol.* 12:37 10.1186/1471-2180-12-37 PMC3340321 22429905

[B18] Cantas L. Sørby J. R. Aleström P. Sørum H. (2012b). Culturable gut microbiota diversity in zebrafish. *Zebrafish* 9 26–37. 10.1089/zeb.2011.0712 22428747 PMC3308716

[B19] Cao Y. He S. Zhou Z. Zhang M. Mao W. Zhang H. (2012). Orally administered thermostable N-acyl homoserine lactonase from *Bacillus* sp. strain AI96 attenuates *Aeromonas hydrophila* infection in zebrafish. *Appl. Environ. Microbiol.* 78 1899–1908. 10.1128/AEM.06139-11 22247159 PMC3298178

[B20] Chen P. L. Wu C. J. Tsai P. J. Tang H. J. Chuang Y. C. Lee N. Y. (2014). Virulence diversity among bacteremic *Aeromonas* isolates: ex vivo, animal, and clinical evidences. *PLoS ONE* 9:e111213 10.1371/journal.pone.0111213 PMC4222899 25375798

[B21] Clatworthy A. E. Lee J. S. Leibman M. Kostun Z. Davidson A. J. Hung D. T. (2009). *Pseudomonas aeruginosa* infection of zebrafish involves both host and pathogen determinants. *Infect. Immun.* 77 1293–1303. 10.1128/IAI.01181-08 19168742 PMC2663173

[B22] Colucci-Guyon E. Tinevez J. Y. Renshaw S. A. Herbomel P. (2011). Strategies of professional phagocytes in vivo: unlike macrophages, neutrophils engulf only surface-associated microbes. *J. Cell Sci.* 124 3053–3059. 10.1242/jcs.082792 21868367

[B23] Cui C. Benard E. L. Kanwal Z. Stockhammer O. W. Van der Vaart M. Zakrzewska A. (2011). “Infectious disease modeling and innate immune function in zebrafish embryos,” in *Methods in Cell Biology* 105 *The Zebrafish: Diseases Models and Chemical Screens*, eds Detrich H. W. Westerfield M. Zon L. I. (Waltham, MA: Academic Press) 274–302. 10.1016/B978-0-12-381320-6.00012-6 21951535

[B24] Deng Q. Huttenlocher A. (2012). Leukocyte migration from a fish eye’s view. *J. Cell Sci.* 125 3949–3956. 10.1242/jcs.093633 23104739 PMC3482313

[B25] Deng Q. Sarris M. Bennin D. A. Green J. M. Herbomel P. Huttenlocher A. (2013). Localized bacterial infection induces systemic activation of neutrophils through Cxcr2 signaling in zebrafish. *J. Leukoc. Biol.* 93 761–769. 10.1189/jlb.1012534 23475575 PMC4050646

[B26] Dinarello C. A. (2009). Immunological and inflammatory functions of the interleukin-1 family. *Annu. Rev. Immunol.* 27 519–550. 10.1146/annurev.immunol.021908.132612 19302047

[B27] Dios S. Romero A. Chamorro R. Figueras A. Novoa B. (2010). Effect of the temperature during antiviral immune response ontogeny in teleosts. *Fish Shellfish Immunol.* 29 1019–1027. 10.1016/j.fsi.2010.08.006 20728541

[B28] Elks P. M. Brizee S. van der Vaart M. Walmsley S. R. van Eeden F. J. Renshaw S. A. (2013). Hypoxia inducible factor signaling modulates susceptibility to mycobacterial infection via a nitric oxide dependent mechanism. *PLoS Pathog.* 9:e1003789 10.1371/journal.ppat.1003789 PMC3868520 24367256

[B29] Ellett F. Lieschke G. J. (2012). Computational quantification of fluorescent leukocyte numbers in zebrafish embryos. *Methods Enzymol.* 506 425–435. 10.1016/B978-0-12-391856-7.00046-9 22341237

[B30] Eming S. A. Krieg T. Davidson J. M. (2007). Inflammation in wound repair: molecular and cellular mechanisms. *J. Invest. Dermatol.* 127 514–525. 10.1038/sj.jid.5700701 17299434

[B31] Fadl A. A. Galindo C. L. Sha J. Zhang F. Garner H. R. Wang H. Q. (2007). Global transcriptional responses of wild-type *Aeromonas hydrophila* and its virulence-deficient mutant in a murine model of infection. *Microb. Pathog.* 42 193–203. 10.1016/j.micpath.2007.02.002 17368824

[B32] Feng D. Marshburn D. Jen D. Weinberg R. J. Taylor R. M. II Burette A. (2007). Stepping into the third dimension. *J. Neurosci.* 27 12757–12760. 10.1523/JNEUROSCI.2846-07.2007 18032646 PMC6673302

[B33] Goldsmith J. R. Jobin C. (2012). Think small: zebrafish as a model system of human pathology. *J. Biomed. Biotechnol.* 2012:817341 10.1155/2012/817341 PMC3371824 22701308

[B34] Grunwald D. J. Eisen J. S. (2002). Headwaters of the zebrafish-emergence of a new model vertebrate. *Nat. Rev. Genet.* 3 717–724. 10.1038/nrg892 12209146

[B35] Hall C. Flores M. V. Crosier K. Crosier P. (2009). Live cell imaging of zebrafish leukocytes. *Methods Mol. Biol.* 546 255–271. 10.1007/978-1-60327-977-2_16 19378109

[B36] Hall C. J. Boyle R. H. Astin J. W. Flores M. V. Oehlers S. H. Sanderson L. E. (2013). Immunoresponsive gene 1 augments bactericidal activity of macrophage-lineage cells by regulating β-oxidation-dependent mitochondrial ROS production. *Cell Metab.* 18 265–278. 10.1016/j.cmet.2013.06.018 23931757

[B37] Harikrishnan R. Balasundarama C. (2005). Modern trends in *Aeromonas hydrophila* disease management with fish. *Rev. Fish. Sci.* 13 281–320. 10.1080/10641260500320845

[B38] Herbomel P. Thisse B. Thisse C. (1999). Ontogeny and behaviour of early macrophages in the zebrafish embryo. *Development* 126 3735–3745. 10433904 10.1242/dev.126.17.3735

[B39] Huiting L. N. Laroche F. Feng H. (2015). The zebrafish as a tool to cancer drug discovery. *Austin J. Pharmacol. Ther.* 3:1069. PMC4731041 26835511

[B40] Igbinosa I. H. Igumbor E. U. Aghdasi F. Tom M. Okoh A. I. (2012). Emerging *Aeromonas* species infections and their significance in public health. *Sci. World J.* 2012:625023 10.1100/2012/625023 PMC3373137 22701365

[B41] Isogai S. Horiguchi M. Weinstein B. M. (2001). The vascular anatomy of the developing zebrafish: an atlas of embryonic and early larval development. *Dev. Biol.* 230 278–301. 10.1006/dbio.2000.9995 11161578

[B42] Janda J. M. Abbott S. L. (2010). The genus *Aeromonas*: taxonomy, pathogenicity, and infection. *Clin. Microbiol. Rev.* 23 35–73. 10.1128/CMR.00039-09 20065325 PMC2806660

[B43] Khajanchi B. K. Kirtley M. L. Brackman S. M. Chopra A. K. (2011). Immunomodulatory and protective roles of quorum-sensing signaling molecules N-acyl homoserine lactones during infection of mice with *Aeromonas hydrophila*. *Infect. Immun.* 79 2646–2657. 10.1128/IAI.00096-11 21536794 PMC3191994

[B44] Kimmel C. B. Ballard W. W. Kimmel S. R. Ullmann B. Schilling T. F. (1995). Stages of embryonic development of the zebrafish. *Dev. Dyn.* 203 253–310. 10.1002/aja.1002030302 8589427

[B45] Ko W. C. Chiang S. R. Yan J. J. Chuang Y. C. (2005). Comparative pathogenicity of bacteraemic isolates of *Aeromonas hydrophila* and *Klebsiella pneumoniae*. *Clin. Microbiol. Infect.* 11 553–558. 10.1111/j.1469-0691.2005.01181.x 15966973

[B46] Kolaczkowska E. Kubes P. (2013). Neutrophil recruitment and function in health and inflammation. *Nat. Rev. Immunol.* 13 159–175. 10.1038/nri3399 23435331

[B47] Lam S. H. Chua H. L. Gong Z. Lam T. J. Sin Y. M. (2004). Development and maturation of the immune system in zebrafish, *Danio rerio*: a gene expression profiling, in situ hybridization and immunological study. *Dev. Comp. Immunol.* 28 9–28. 12962979 10.1016/s0145-305x(03)00103-4

[B48] Le Guyader D. Redd M. J. Colucci-Guyon E. Murayama E. Kissa K. Briolat V. (2008). Origins and unconventional behavior of neutrophils in developing zebrafish. *Blood* 111 132–141. 10.1182/blood-2007-06-095398 17875807

[B49] Lesley R. Ramakrishnan L. (2008). Insights into early mycobacterial pathogenesis from the zebrafish. *Curr. Opin. Microbiol.* 11 277–283. 10.1016/j.mib.2008.05.013 18571973 PMC3071758

[B50] Levraud J. P. Colucci-Guyon E. Redd M. J. Lutfalla G. Herbomel P. (2008). In vivo analysis of zebrafish innate immunity. *Methods Mol. Biol.* 415 337–363. 10.1007/978-1-59745-570-1_20 18370164

[B51] Levraud J. P. Disson O. Kissa K. Bonne I. Cossart P. Herbomel P. (2009). Real-time observation of *Listeria monocytogenes* phagocyte interactions in living zebrafish larvae. *Infect. Immun.* 77 3651–3660. 10.1128/IAI.00408-09 19546195 PMC2738018

[B52] Li Y. J. Hu B. (2012). Establishment of multi-site infection model in zebrafish larvae for studying *Staphylococcus aureus* infectious disease. *J. Genet. Genomics* 39 521–534. 10.1016/j.jgg.2012.07.006 23021551

[B53] Lieschke G. J. Currie P. D. (2007). Animal models of human disease: zebrafish swim into view. *Nat. Rev. Genet.* 8 353–367. 10.1038/nrg2091 17440532

[B54] Lieschke G. J. Oates A. C. Crowhurst M. O. Ward A. C. Layton J. E. (2001). Morphologic and functional characterization of granulocytes and macrophages in embryonic and adult zebrafish. *Blood* 98 3087–3096. 10.1182/blood.V98.10.3087 11698295

[B55] Lin C. Y. Chiang C. Y. Tsai H. J. (2016). Zebrafish and Medaka: new model organisms for modern biomedical research. *J. Biomed. Sci.* 23:19 10.1186/s12929-016-0236-5 PMC4730764 26822757

[B56] Liu L. Gong Y. X. Zhu B. Liu G. L. Wang G. X. Ling F. (2015). Effect of a new recombinant *Aeromonas hydrophila* vaccine on the grass carp intestinal microbiota and correlations with immunological responses. *Fish Shellfish Immunol.* 45 175–183. 10.1016/j.fsi.2015.03.043 25862971

[B57] Lü A. J. Hu X. C. Wang Y. Zhu A. H. Shen L. L. Tian J. (2015). Skin immune response in the zebrafish, *Danio rerio* (Hamilton), to *Aeromonas hydrophila* infection: a transcriptional profiling approach. *J. Fish Dis.* 38 137–150. 10.1111/jfd.12214 24517469

[B58] Lugo-Villarino G. Balla K. M. Stachura D. L. Bañuelos K. Werneck M. B. Traver D. (2010). Identification of dendritic antigen-presenting cells in the zebrafish. *Proc. Natl. Acad. Sci. U.S.A.* 107 15850–15855. 10.1073/pnas.1000494107 20733076 PMC2936643

[B59] MacRae C. A. Peterson R. T. (2015). Zebrafish as tools for drug discovery. *Nat. Rev. Drug Discov.* 14 721–731. 10.1038/nrd4627 26361349

[B60] Mantovani A. Cassatella M. A. Costantini C. Jaillon S. (2011). Neutrophils in the activation and regulation of innate and adaptive immunity. *Nat. Rev. Immunol.* 11 519–531. 10.1038/nri3024 21785456

[B61] McCoy A. J. Koizumi Y. Toma C. Higa N. Dixit V. Taniguchi S. (2010). Cytotoxins of the human pathogen *Aeromonas hydrophila* trigger, via the NLRP3 inflammasome, caspase-1 activation in macrophages. *Eur. J. Immunol.* 40 2797–2803. 10.1002/eji.201040490 20722078

[B62] Meijer A. H. (2016). Protection and pathology in TB: learning from the zebrafish model. *Semin. Immunopathol.* 38 261–273. 10.1007/s00281-015-0522-4 26324465 PMC4779130

[B63] Meijer A. H. Spaink H. P. (2011). Host-pathogen interactions made transparent with the zebrafish model. *Curr. Drug Targets* 12 1000–1017. 10.2174/138945011795677809 21366518 PMC3319919

[B64] Meijer A. H. van der Vaart M. Spaink H. P. (2014). Real-time imaging and genetic dissection of host-microbe interactions in zebrafish. *Cell Microbiol.* 16 39–49. 10.1111/cmi.12236 24188444

[B65] Mertz P. M. Patti J. Marcin J. Marshall D. (1987). Model for studying bacterial adherence to skin wounds. *J. Clin. Microbiol.* 25 1601–1604. 3116035 10.1128/jcm.25.9.1601-1604.1987 PMC269291

[B66] Mesureur J. Vergunst A. C. (2014). Zebrafish embryos as a model to study bacterial virulence. *Methods Mol. Biol.* 1197 41–66. 10.1007/978-1-4939-1261-2_3 25172274

[B67] Mostowy S. Boucontet L. Mazon Moya M. J. Sirianni A. Boudinot P. Hollinshead M. (2013). The zebrafish as a new model for the in vivo study of *Shigella flexneri* interaction with phagocytes and bacterial autophagy. *PLoS Pathog.* 9:e1003588 10.1371/journal.ppat.1003588 PMC3764221 24039575

[B68] Myllymäki H. Bäuerlein C. A. Rämet M. (2016). The zebrafish breathes new life into the study of Tuberculosis. *Front. Immunol.* 19:196 10.3389/fimmu.2016.00196 PMC4871865 27242801

[B69] Neely M. N. Pfeifer J. D. Caparon M. (2002). Streptococcus-zebrafish model of bacterial pathogenesis. *Infect. Immun.* 70 3904–3914. 10.1128/IAI.70.7.3904-3914.2002 12065534 PMC128100

[B70] Nguyen-Chi M. Phan Q. T. Gonzalez C. Dubremetz J. F. Levraud J. P. Lutfalla G. (2014). Transient infection of the zebrafish notochord with *E. coli* induces chronic inflammation. *Dis. Model Mech.* 7 871–882. 10.1242/dmm.014498 24973754 PMC4073276

[B71] Novoa B. Figueras A. (2012). Zebrafish: model for the study of inflammation and the innate immune response to infectious diseases. *Adv. Exp. Med. Biol.* 946 253–275. 10.1007/978-1-4614-0106-3_15 21948373

[B72] Nowik N. Podlasz P. Jakimiuk A. Kasica N. Sienkiewicz W. Kaleczyc J. (2015). Zebrafish: an animal model for research in veterinary medicine. *Pol. J. Vet. Sci.* 18 663–674. 10.1515/pjvs-2015-0086 26618602

[B73] Ogryzko N. V. Hoggett E. E. Solaymani-Kohal S. Tazzyman S. Chico T. J. Renshaw S. A. (2014). Zebrafish tissue injury causes upregulation of interleukin-1 and caspase-dependent amplification of the inflammatory response. *Dis. Model Mech.* 7 259–264. 10.1242/dmm.013029 24203886 PMC3917246

[B74] Ordas A. Raterink R. J. Cunningham F. Jansen H. J. Wiweger M. I. Jong-Raadsen S. (2015). Testing tuberculosis drug efficacy in a zebrafish high-throughput translational medicine screen. *Antimicrob. Agents Chemother.* 59 753–762. 10.1128/AAC.03588-14 25385118 PMC4335901

[B75] O’Toole R. Von Hofsten J. Rosqvist R. Olsson P. E. Wolf-Watz H. (2004). Visualisation of zebrafish infection by GFP-labelled *Vibrio anguillarum*. *Microb. Pathog.* 37 41–46. 10.1016/j.micpath.2004.03.001 15194159

[B76] Pardo-Martin C. Allalou A. Medina J. Eimon P. M. Wählby C. Fatih Yanik M. (2013). High-throughput hyperdimensional vertebrate phenotyping. *Nat. Commun.* 4:1467 10.1038/ncomms2475 PMC3573763 23403568

[B77] Parker J. L. Shaw J. G. (2011). *Aeromonas* spp. clinical microbiology and disease. *J. Infect.* 62 109–118. 10.1016/j.jinf.2010.12.003 21163298

[B78] Pfaﬄ M. W. (2001). A new mathematical model for relative quantification in real-time RT-PCR. *Nucleic Acids Res.* 29 2002–2007. 10.1093/nar/29.9.e45 PMC55695 11328886

[B79] Phillips J. B. Westerfield M. (2014). Zebrafish models in translational research: tipping the scales toward advancements in human health. *Dis. Model Mech.* 7 739–743. 10.1242/dmm.015545 24973743 PMC4073263

[B80] Poobalane S. Thompson K. D. Ardó L. Verjan N. Han H. J. Jeney G. (2010). Production and efficacy of an *Aeromonas hydrophila* recombinant S-layer protein vaccine for fish. *Vaccine* 28 3540–3547. 10.1016/j.vaccine.2010.03.011 20307596

[B81] Prajsnar T. Cunliffe V. J. Foster S. Renshaw S. A. (2008). A novel vertebrate model of *Staphylococcus aureus* infection reveals phagocyte-dependent resistance of zebrafish to non-host specialized pathogens. *Cell. Microbiol.* 10 2312–2325. 10.1111/j.1462-5822.2008.01213.x 18715285

[B82] Pressley M. Phelan P. Witten P. Mellon M. Kim C. (2005). Pathogenesis and inflammatory response to *Edwardsiella tarda* infection in the zebrafish. *Dev. Comp. Immunol.* 29 501–513. 10.1016/j.dci.2004.10.007 15752547

[B83] Renshaw S. A. Loynes C. A. Trushell D. M. Elworthy S. Ingham P. W. Whyte M. K. (2006). A transgenic zebrafish model of neutrophilic inflammation. *Blood* 108 3976–3978. 10.1182/blood-2006-05-024075 16926288

[B84] Renshaw S. A. Trede N. S. (2012). A model 450 million years in the making: zebrafish and vertebrate immunity. *Dis. Model Mech.* 5 38–47. 10.1242/dmm.007138 22228790 PMC3255542

[B85] Rodríguez I. Chamorro R. Novoa B. Figueras A. (2009). b-Glucan administration enhances disease resistance and some innate immune responses in zebrafish (*Danio rerio*). *Fish Shellfish Immunol.* 27 369–373. 10.1016/j.fsi.2009.02.007 19232393

[B86] Rodríguez I. Novoa B. Figueras A. (2008). Immune response of zebrafish (*Danio rerio*) against a newly isolated bacterial pathogen *Aeromonas hydrophila*. *Fish Shellfish Immunol.* 25 239–249. 10.1016/j.fsi.2008.05.002 18640853

[B87] Rounioja S. Saralahti A. Rantala L. Parikka M. Henriques-Normark B. Silvennoinen O. (2012). Defense of zebrafish embryos against *Streptococcus pneumoniae* infection is dependent on the phagocytic activity of leukocytes. *Dev. Comp. Immunol.* 36 342–348. 10.1016/j.dci.2011.05.008 21658407

[B88] Rozen S. Skaletsky H. (2000). Primer3 on the WWW for general users and for biologist programmers. *Methods Mol. Biol.* 132 365–386. 10547847 10.1385/1-59259-192-2:365

[B89] Santoriello C. Zon L. I. (2012). Hooked! Modeling human disease in zebrafish. *J. Clin. Invest.* 122 2337–2343. 10.1172/JCI60434 22751109 PMC3386812

[B90] Sha J. Pillai L. Fadl A. A. Galindo C. L. Erova T. E. Chopra A. K. (2005). The type III secretion system and cytotoxic enterotoxin alter the virulence of *Aeromonas hydrophila*. *Infect. Immun.* 73 6446–6457. 10.1128/IAI.73.10.6446-6457.2005 16177316 PMC1230953

[B91] Sha J. Wang S. F. Suarez G. Sierra J. C. Fadl A. A. Erova T. E. (2007). Further characterization of a type III secretion system (T3SS) and of a new effector protein from a clinical isolate of *Aeromonas hydrophila*–part I. *Microb. Pathog.* 43 127–146. 10.1016/j.micpath.2007.05.002 17644303

[B92] Spaink H. P. Cui C. Wiweger M. I. Jansen H. J. Veneman W. J. Marín-Juez R. (2013). Robotic injection of zebrafish embryos for high-throughput screening in disease models. *Methods* 62 246–254. 10.1016/j.ymeth.2013.06.002 23769806

[B93] Suarez G. Sierra J. C. Kirtley M. L. Chopra A. K. (2010). Role of Hcp, a type 6 secretion system effector, of *Aeromonas hydrophila* in modulating activation of host immune cells. *Microbiology* 156 3678–3688. 10.1099/mic.0.041277-0 20798163 PMC3068704

[B94] Tobin D. M. May R. C. Wheeler R. T. (2012). Zebrafish: a see-through host and a fluorescent toolbox to probe host-pathogen interaction. *PLoS Pathog.* 8:e1002349 10.1371/journal.ppat.1002349 PMC3252360 22241986

[B95] Tomás J. M. (2012). The main *Aeromonas* pathogenic factors. *ISRN Microbiol.* 2012:256261 10.5402/2012/256261 PMC3658858 23724321

[B96] Torraca V. Cui C. Boland R. Bebelman J. P. van der Sar A. M. Smit M. J. (2015). The CXCR3-CXCL11 signaling axis mediates macrophage recruitment and dissemination of mycobacterial infection. *Dis. Model Mech.* 8 253–269. 10.1242/dmm.017756 25573892 PMC4348563

[B97] Torraca V. Masud S. Spaink H. P. Meijer A. H. (2014). Macrophage-pathogen interactions in infectious diseases: new therapeutic insights from the zebrafish host model. *Dis. Model Mech.* 7 785–797. 10.1242/dmm.015594 24973749 PMC4073269

[B98] Trede N. S. Langenau D. M. Traver D. Look A. T. Zon L. I. (2004). The use of zebrafish to understand immunity. *Immunity* 20 367–379. 10.1016/S1074-7613(04)00084-6 15084267

[B99] van der Sar A. Musters R. van Eeden F. Appelmelk B. Vandenbroucke-Grauls C. Bitter W. (2003). Zebrafish embryos as a model host for the real time analysis of *Salmonella typhimurium* infections. *Cell. Microbiol.* 5 601–611. 10.1046/j.1462-5822.2003.00303.x 12925130

[B100] Van der Vaart M. Spaink H. P. Meijer A. H. (2012). Pathogen recognition and activation of the innate immune response in zebrafish. *Adv. Hematol.* 2012:159807 10.1155/2012/159807 PMC3395205 22811714

[B101] Varela M. Romero A. Dios S. van der Vaart M. Figueras A. Meijer A. H. (2014). Cellular visualization of macrophage pyroptosis and interleukin-1β release in a viral hemorrhagic infection in zebrafish larvae. *J. Virol.* 88 12026–12040. 10.1128/JVI.02056-14 25100833 PMC4178746

[B102] Vergunst A. C. Meijer A. H. Renshaw S. A. O’Callaghan D. (2010). *Burkholderia cenocepacia* creates an intramacrophage replication niche in zebrafish embryos, followed by bacterial dissemination and establishment of systemic infection. *Infect. Immun.* 78 1495–1508. 10.1128/IAI.00743-09 20086083 PMC2849400

[B103] Vilches S. Canals R. Wilhelms M. Saló M. T. Knirel Y. A. Vinogradov E. (2007). Mesophilic *Aeromonas* UDP-glucose pyrophosphorylase (GalU) mutants show two types of lipopolysaccharide structures and reduced virulence. *Microbiology* 153 2393–2404. 10.1099/mic.0.2007/006437-0 17660404

[B104] Volkman H. E. Pozos T. C. Zheng J. Davis J. M. Rawls J. F. Ramakrishnan L. (2010). Tuberculous granuloma induction via interaction of a bacterial secreted protein with host epithelium. *Science* 327 466–469. 10.1126/science.1179663 20007864 PMC3125975

[B105] Wajant H. Pfizenmaier K. Scheurich P. (2003). Tumor necrosis factor signaling. *Cell Death Differ.* 10 45–65. 10.1038/sj.cdd.4401189 12655295

[B106] Westerfield M. (2007). *The Zebrafish Book: A Guide for the Laboratory Use of Zebrafish (Danio rerio)* 5th Edn. Eugene, OR: University of Oregon Press.

[B107] Wong C. Y. Mayrhofer G. Heuzenroeder M. W. Atkinson H. M. Quinn D. M. Flower R. L. (1996). Measurement of virulence of aeromonads using a suckling mouse model of infection. *FEMS Immunol. Med. Microbiol.* 15 233–241. 10.1111/j.1574-695X.1996.tb00089.x 8908484

[B108] Yan B. Han P. Pan L. Lu W. Xiong J. Zhang M. (2014). IL-1β and reactive oxygen species differentially regulate neutrophil directional migration and Basal random motility in a zebrafish injury-induced inflammation model. *J. Immunol.* 192 5998–6008. 10.4049/jimmunol.1301645 24835391

[B109] Yoo S. K. Huttenlocher A. (2011). Spatiotemporal photolabeling of neutrophil trafficking during inflammation in live zebrafish. *J. Leukoc. Biol.* 89 661–667. 10.1189/jlb.1010567 21248150 PMC3079246

[B110] Yu H. B. Rao P. S. Lee H. C. Vilches S. Merino S. Tomas J. M. (2004). A type III secretion system is required for *Aeromonas hydrophila* AH-1 pathogenesis. *Infect. Immun.* 72 1248–1256. 10.1128/IAI.72.3.1248-1256.2004 14977925 PMC356039

[B111] Yu H. B. Zhang Y. L. Lau Y. L. Yao F. Vilches S. Merino S. (2005). Identification and characterization of putative virulence genes and gene clusters in *Aeromonas hydrophila* PPD134/91. *Appl. Environ. Microbiol.* 71 4469–4477. 10.1128/AEM.71.8.4469-4477.2005 16085838 PMC1183340

[B112] Zakrzewska A. Cui C. Stockhammer O. W. Benard E. L. Spaink H. P. Meijer A. H. (2010). Macrophage-specific gene functions in Spi1-directed innate immunity. *Blood* 116 e1–e11. 10.1182/blood-2010-01-262873 20424185

